# Can ecological niche models be used to accurately predict the distribution of invasive insects? A case study of *Hyphantria cunea* in China

**DOI:** 10.1002/ece3.11159

**Published:** 2024-03-14

**Authors:** Xuanye Wen, Guofei Fang, Shouquan Chai, Chuanjie He, Shouhui Sun, Guanghua Zhao, Xiao Lin

**Affiliations:** ^1^ Center for Biological Disaster Prevention and Control National Forestry and Grassland Administration Shenyang China; ^2^ College of Forestry Shenyang Agricultural University Shenyang China; ^3^ College of Life Sciences Shanxi Normal University Taiyuan China

**Keywords:** Biomod2, *Hyphantria cunea*, MaxEnt, niche model, potential distribution

## Abstract

In recent decades, ecological niche models (ENMs) have been widely used to predict suitable habitats for species. However, for invasive organisms, the prediction accuracy is unclear. In this study, we employed the most widely used maximum entropy (MaxEnt) model and ensemble model (EM) Biomod2 and verified the practical effectiveness of the ENM in predicting the distribution areas of invasive insects based on the true occurrence of *Hyphantria cunea* in China. The results showed that when only limited data of invasive areas were used, the two ENMs could not effectively predict the distribution of suitable habitats of *H. cunea*, although the use of global data can greatly improve the prediction accuracy of ENMs. When analyzing the same data, Biomod2's prediction accuracy was significantly better than that of MaxEnt. For long‐term predictions, the area of suitable habitat predicted by the ENMs was much greater than the occurrence area; for short‐term predictions, the accuracy of the predicted area was significantly improved. Under the current conditions, the area of suitable habitat for *H. cunea* in China is 118 × 10^4^ km^2^, of which 59.32% is moderately or highly suitable habitat. Future climate change could significantly increase the suitable habitat area of *H. cunea* in China, and the predicted area of suitable habitats in all climate scenarios exceeded 355 × 10^4^ km^2^, accounting for 36.98% of the total land area in China. This study demonstrates the use of ENMs to study invasive insects and provides a reference for the management of *H. cunea* in China.

## Graphical abstract

This study verifies the accuracy of ENMs in predicting the distribution area of invasive insects. The comprehensiveness of species distribution points is the key to determining whether the results are accurate. The prediction performance of the EM is better than that of the single model.

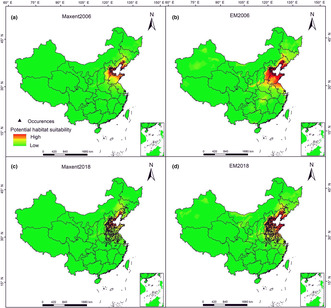

## 1 INTRODUCTION

Invasive organisms are a main driver of biodiversity loss (Hughes, 2017). They not only threaten the stability of ecosystem structure and function but also cause serious damage to normal production in many fields, such as agriculture, forestry, animal husbandry, and fisheries (Cuthbert et al., 2021). As global economic integration continues to accelerate, biological invasion incidents are expected to occur frequently in the next 30 years (Seebens et al., 2021). Insects, as the main species of invasive organisms, have been introduced to various parts of the world through transportation, trade, and tourism. They exhibit traits common to many invasive species, including rapid proliferation and the ability to cause significant damage (Kenis et al., 2009; McLaughlin & Dearden, 2019). Although many countries worldwide have adopted measures such as introducing legislation and promoting public awareness to curb biological invasions (Barker et al., 2020; Garnas et al., 2016), resisting insect invasions and curbing their spread remain major challenges in pest management (Wan et al., 2017).

Ecological niche models (ENMs) belong to a category of models that seek to elucidate and forecast species distribution patterns by incorporating ecological principles such as equilibrium in species distribution, habitat saturation, niche conservatism, unrestricted dispersal capabilities, and the perceived limited influence of biotic interactions on shaping extensive species distributions (Schickele et al., 2020; Soberon & Peterson, 2005; Wiens et al., 2009). These models aim to capture and analyze the connections between species and their environments (Hao et al., 2020). Using ENMs to predict potential suitable habitats for invasive insects has many obvious benefits, such as the introduction of targeted biological predators and the facilitate of management decisions. In recent years, many studies on invasive insects have utilized ENMs. Ramasamy et al. (2022) used the maximum entropy (MaxEnt) model to predict the impact of future climate change on the global distribution of *Spodoptera frugiperda*; Finch et al. (2021) used CLIMEX to assess the global potential distribution of *Paracoccus marginatus*; and Herrera et al. (2023) analyzed the invasion risk of *Vespa velutina* on Mediterranean islands using an ensemble of small models (EM).

However, there are more than 30 ENMs (Norberg et al., 2019), and these models use different methods (Elith et al., 2011). The results are not consistent, and incorrect results could misinform managers making policy decisions, thus having a serious negative impact on the effective prevention of biological invasions (Saupe et al., 2012). Therefore, whether ENMs can accurately predict the distribution of invasive insects is unclear. Establishing whether the results obtained by different models are correct and whether the use of a limited sample of indigenous data affects the results are questions worthy of discussion.

**FIGURE 1:**
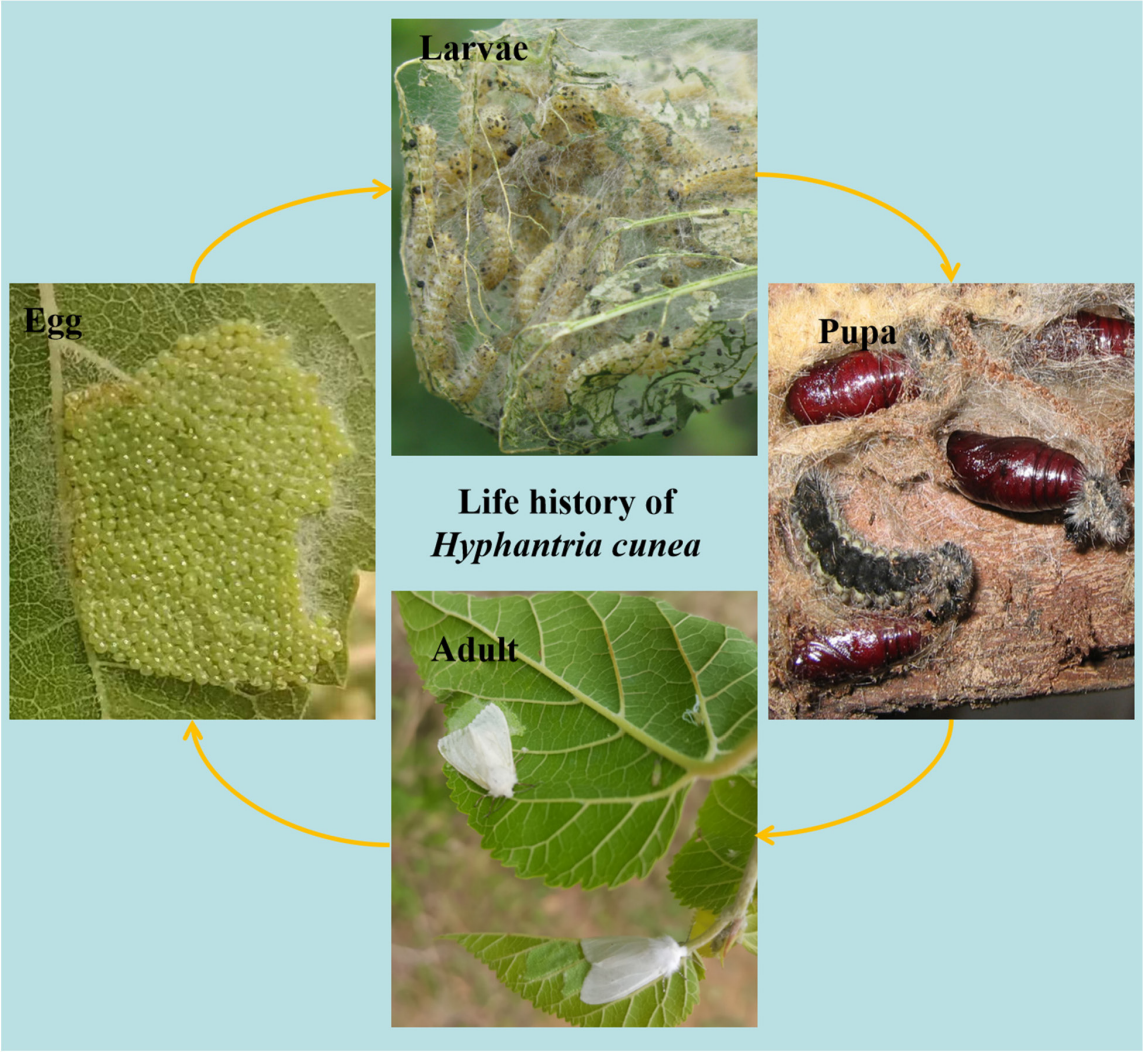
Life history of the fall webworm (*Hyphantria cunea*), Liaoning, China (Photo credit: Shouhui Sun).

The fall webworm (*Hyphantria cunea*) is a notorious invasive insect (Figure 1). In its native North America, *H. cunea* does not cause serious harm, but in Asia and Europe, *H. cunea* has exhibited amazing destructive power (Wu et al., 2019). *Hyphantria cunea* can feed on more than 600 kinds of plants (Chen et al., 2020). Even more concerning is the extremely strong reproductive ability of *H. cunea*; a single fall webworm moth can produce nearly 200 million offspring in 1 year (Yang et al., 2006). In addition, *H. cunea* is very resilient; a high‐temperature and high‐humidity environment does not have a large effect on the survival of *H. cunea* (Xu et al., 2015). In China, *H. cunea* is regarded as a strictly managed invasive organism and a quarantine pest (Yang et al., 2014; Zhang et al., 2023); thus, the Chinese central government has issued three orders to increase the management of *H. cunea*, and local governments at all levels are required to report annually on the occurrence of *H. cunea*. Therefore, compared with those of other invasive insects, the data on the occurrence of *H. cunea* in China are extremely accurate and detailed, which is very helpful for verifying the accuracy of the species distribution model for predicting invasive insects.

Several studies have been conducted to predict the distribution of *H. cunea* in China using ENMs (Ge et al., 2019; Tang et al., 2021); however, all these studies adopted a single model and lacked comparisons of the actual effects of different models. In this paper, the most widely used ENM, MaxEnt, and EM, Biomod2, were used. Based on the distribution of *H. cunea* in China and globally, we explored the effects of different models and sample data on model prediction results, and the optimal model was used to predict the distribution of *H. cunea* suitable habitat in China in current and future climates. This research can help to promote the correct application of ENMs in the prevention and control of invasive insects and can provide scientific guidance for the control of *H. cunea* in China.

## 2 MATERIALS AND METHODS

### 2.1 Occurrence and environmental data

In this study, data on the distribution of two *H. cunea* populations in China and globally were used for modeling analysis. The data in China were from the Announcements of *H. cunea* Infested Areas in China (http://www.forestry.gov.cn), published by the National Forestry and Grassland Administration in 2007, 2019, and 2022, and these data represent the true distribution of *H. cunea* in the administrative regions above the county level in China in 2006 (the year of the first announcement), 2018 (the latest data in the WorldClim database), and 2021 (the year of the latest announcement), with 125, 592, and 611 data points, respectively. For the global distribution, the report of each corresponding year published by the Global Biodiversity Information Facility (http://www.gbif.org/) was selected, records were screened, nonnatural records were removed, and redundant data were deleted. To reduce the error caused by the clustering effect, each grid (5 × 5 km) retained only one distribution point.

In this study, we used 22 environmental variables of climate and topography for modeling, and all data were downloaded from the WorldClim database (http://worldclim.org/data/index.html). Future distribution models were developed using general circulation models (GCMs) with different climate sensitivities: ACCESS‐CM2 (Commonwealth Scientific and Industrial Research Organization and Australian Research Council Centre of Excellence for Climate System Science), BCC‐CSM2‐MR (Beijing Climate Center), CMCC‐ESM2 (Fondazione Centro Euro‐Mediterraneo sui Cambiamenti Climatici), IPSL‐CM6A‐LR (Institut Pierre Simon Laplace), and MIROC6 (Ibaraki Research Institute for Environmental Studies). Future climate projections were developed for 2030 and 2050. Three future climate scenarios (SSP126, SSP245, and SSP585) were utilized, representing low, medium, and high greenhouse gas emission scenarios, respectively. The spatial resolution of each factor was set to 2.5 m″ (approximately 20 km^2^; Graham, 2003).

To avoid overfitting of the model prediction results due to the collinearity among various environmental factors, *R* was used for the screening of the environmental variables according to the variance inflation factor (VIF) and the Spearman correlation test, thus improving the accuracy of the ENM by reducing the complexity of the model. The factors with a correlation less than 0.7 were screened, and on this basis, the factors with a VIF less than 5 were selected; additionally, all factors with a Spearman correlation coefficient less than 0.7 were selected, and for factors with a Spearman correlation coefficient greater than 0.7, only those with a high ecological significance were selected (Rose, 1995; Zhao et al., 2021). Ultimately, 10 environmental variables were included (Table S1).

### 2.2 Ecological niche modeling

Since our models compare the environment at occurrence localities to the environment at background localities, we need to sample random points from a background extent. Using MaxEnt's settings instruction, the maximum number of background points was set to 10,000, and the running property was set to repeat five times. For each run, 75% of the distribution samples were randomly selected for use as the training set, and the remaining 25% were used as the test dataset to validate the model. Bootstrapping was conducted, the results were output in logistic format, and the average of the five simulation results was selected to build the MaxEnt model.

Biomod2 can create an EM based on species distribution data, pseudodistribution data, and pseudoabsence points generated by the BIOMOD_FormatingData function, and the random command was used to randomly generate 80,000 pseudoexistence data points for model simulation. The Biomod‐tuning command was applied to optimize the model parameters, 75% of the sample data were selected as the training set, the weights of the distribution data and the pseudodistribution data were set to be equal, the process was repeated five times, 50 simulation models were generated, and single models with a true skill statistic (TSS) ≥ 0.7 were selected. An EM was constructed to simulate the potential distribution area of *H. cunea* using the weighted average method.

### 2.3 Accuracy evaluation of the model

In addition to using the area under the receiver operating characteristic (ROC) curve (AUC), TSS, kappa, and other common evaluation metrics (Wen et al., 2022), a true occurrence comparison method was employed; that is, the results predicted by the model were compared with the occurrence data of *H. cunea*, and the closer the comparison was to 100%, the higher the model prediction accuracy was. Here, we arbitrarily set a comparison threshold of 75%, and the next comparison is only performed if the prediction area contains more than 75% of the actual occurring points, while those that do not satisfy this condition are judged to be inaccurately predicted by the model.

The steps of the comparison are as follows:Using the data from 2006, the prediction results of the MaxEnt and EM models for 2018 were obtained and compared with the occurrence data; if the occurrence rate exceeded the threshold, then the 2018 data were used to predict the distribution of suitable habitats in 2021, and the results were compared with the occurrence data. When the occurrence rate of the model point was high, the ratio of the predicted area to the occurrence area was used as a secondary indicator, and the closer the ratio was to 1, the better the model accuracy. If the occurrence rate of the model point was less than the threshold, there was no need to calculate the ratio.

### 2.4 Current and future suitable distribution areas of the optimal model

The optimal model obtained by the evaluation was used to model the current suitable distribution area of *H. cunea*. By entering the bm_FindOptimStat function to obtain the presence/absence (0/1) cutoff, the area with a value lower than the cutoff was an unsuitable area, and the areas with a value higher than the cutoff were divided into three equal parts, corresponding to the low, moderate and high suitability areas. In ArcGIS, the distribution change between the binary species distribution modeling (SDM) tool in the plug‐in SDM tools was used to calculate the area change.

The future climate scenarios were imported into the optimal model, the presence/absence (0, 1) matrix of species distribution in each spatial unit was obtained and compared with the presence/absence (0, 1) matrix of the current suitable area. The average of the five GCMs results was loaded into ArcGIS (Wei et al., 2019), and a raster calculator was used to derive the spatial pattern of changes in the fitness zone of *H. cunea* under future climate change scenarios.

## 3 RESULTS

### 3.1 China occurrence data model

**FIGURE 2:**
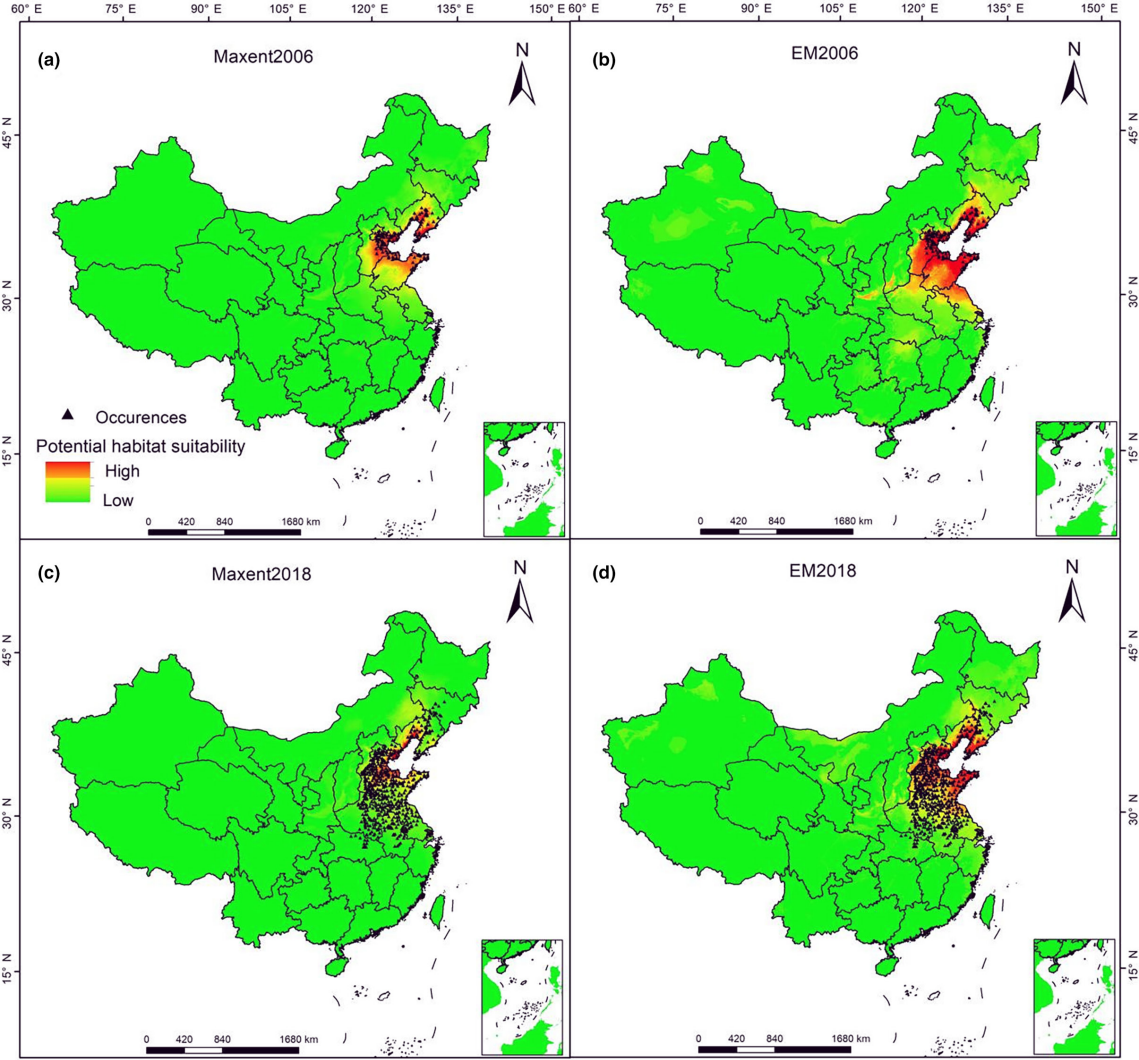
Distribution of suitable habitats for *Hyphantria cunea* using the 2006 data in China. The black triangle points in the figure represent the actual occurrence points of *H. cunea* in the current year. (a, c) are the prediction results of MaxEnt model. (b, d) are the prediction results of Biomod2. The color from orange to green indicates that the potential habitat suitability is from high to low.

Figure 2 shows the prediction results of the ENM constructed using the distribution data of *H. cunea* in China in 2007. The ranges of suitable habitats predicted by the EM in the current year and in 2018 were larger than those predicted by MaxEnt. Although the AUC, TSS, kappa, and other evaluation indicators all indicated that the model results were excellent (Figure S1), the prediction accuracy of MaxEnt was only 3.72%, whereas Biomod2 exhibited a significantly higher accuracy at 53.04%. The predictions of both models were below the preset accuracy thresholds, and neither well reflected the occurrence of *H. cunea* in China in 2018. There are many occurrence areas in central and eastern China that were not included in the prediction results, and these areas were mainly characterized by lower latitudes and higher average temperature and precipitation than those of the occurrence areas identified before 2007.

### 3.2 Global occurrence data model

Compared with the Chinese occurrence data model, the prediction accuracy of the suitable habitat model based on the global distribution points of *H. cunea* was significantly improved (Table 1); the prediction accuracy of MaxEnt was 79.05%, the accuracy of Biomod2 reached 95.44%, and both values were larger than the preset accuracy threshold. However, the area obtained by the model far exceeded the actual occurrence area of *H. cunea*. The predicted areas of MaxEnt and Biomod2 were 4.7 times and 5.9 times the occurrence area, respectively.

**TABLE 1:** The prediction accuracy of the two models using different data.

Model	Data type	Predicted points	Real points	Percentage (%)	Predicted area (×10^4^ km^2^)	Occurred area (×10^4^ km^2^)	Percentage (%)
MaxEnt	2006‐China	22	592	3.72			
Biomod2	2006‐China	314	592	53.04			
MaxEnt	2006‐Global	468	592	79.05	400	86	465.12
Biomod2	2006‐Global	565	592	95.44	511	86	594.19
MaxEnt	2018‐Global	605	611	99.02	222	92	241.30
Biomod2	2018‐Global	611	611	100.00	118	92	128.26

**FIGURE 3:**
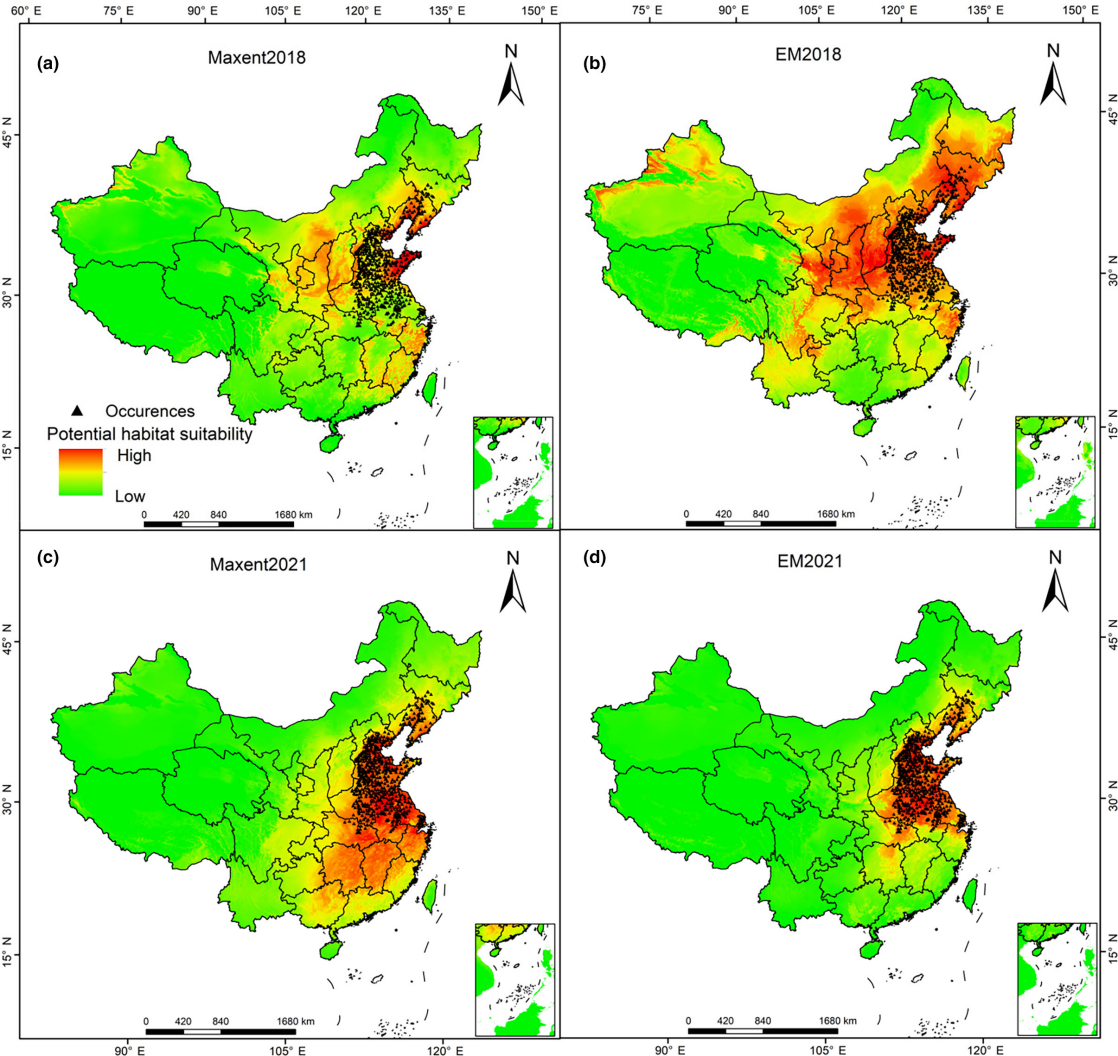
Distribution of suitable habitats of *Hyphantria cunea* using global data. The black triangle points in the figure represent the actual occurrence points of *H. cunea* in the current year. (a, c) are the prediction results of the MaxEnt model. (b, d) are the prediction results of Biomod2. The color from orange to green indicates potential habitat suitability from high to low.

Figure 3 shows the two models constructed based on the global data from 2018. Compared with the occurrence data from 2021, the prediction accuracy of Biomod2 reached an impressive 100%, and the accuracy of MaxEnt reached 99.02%, with only 6 unpredicted occurrence points. The predicted areas of the two models were also more consistent with the occurred area. The predicted area of Biomod2 was only 28.26% more than the actual area, and the predicted area of MaxEnt was only 2.4 times the actual area (a decrease from 4.7 times in 2018). In terms of short‐term predictions, the two models achieved good results.

### 3.3 The current distribution of suitable habitats of *Hyphantria cunea* in China

**FIGURE 4:**
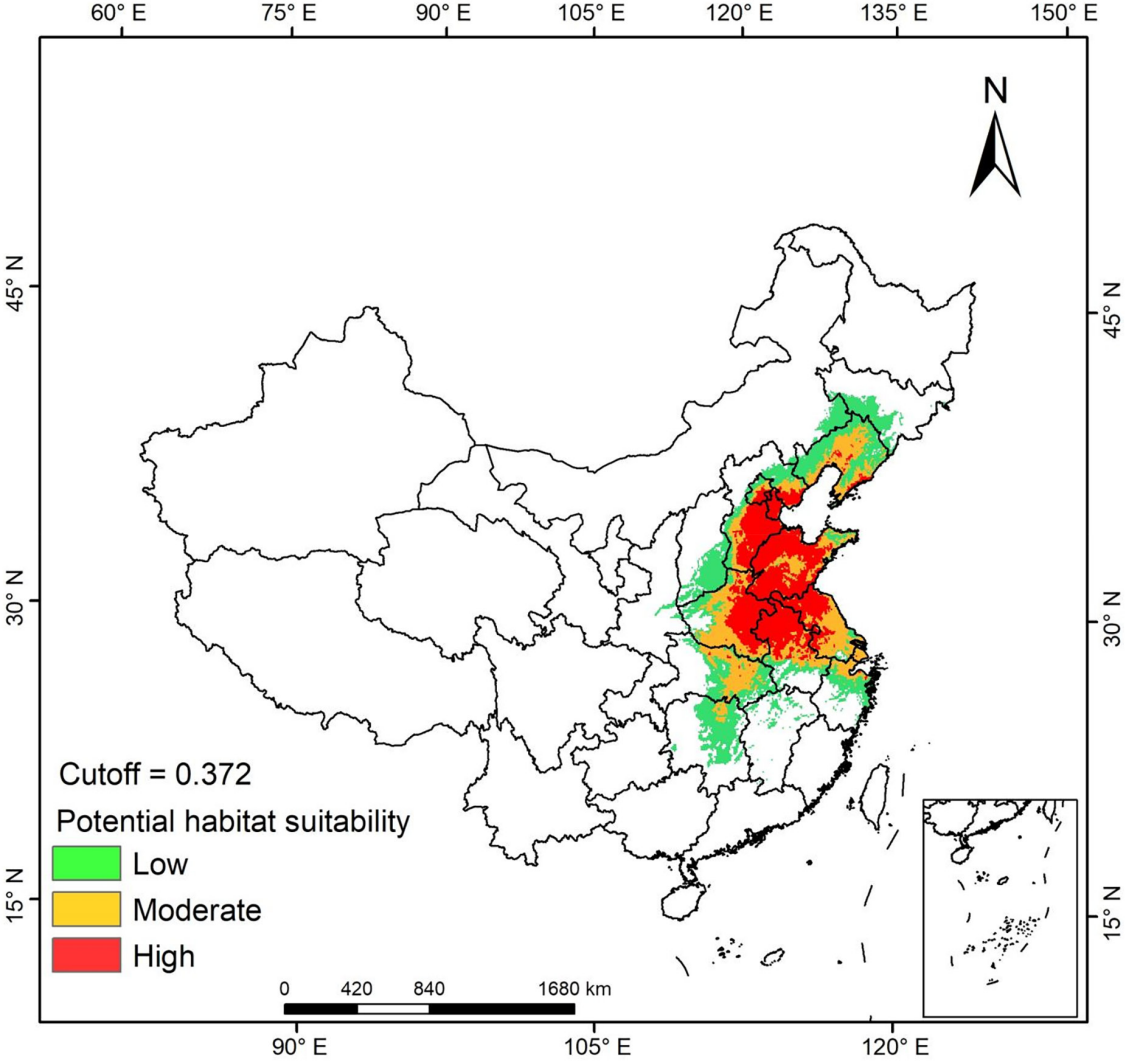
The current suitable distribution range of *Hyphantria cunea*. The areas with a value higher than the cutoff were divided into three equal parts corresponding to the low, moderate, and high suitability areas.

Biomod2, with a prediction accuracy of 100%, was used to predict the current distribution of suitable habitats (Figure 4). The results show that the area of highly suitable habitat in China was 37 × 10^4^ km^2^, accounting for 31.27% of all predicted areas, and highly suitable habitats were located mainly in Central China and East China, which have the most serious *H. cunea* invasions in China. The area of moderately suitable habitat was 33 × 10^4^ km^2^, accounting for 27.95% of all predicted areas, and moderately suitable habitats were located mainly in the eastern coastal area, along the Yangtze River and on the plains in Northeast China, with most of the new occurrences of *H. cunea* occurring in these regions in the last 5 years. The area of low habitat suitability was 48 × 10^4^ km^2^, with the northernmost area reaching 43° N latitude, the southernmost area reaching 26° N latitude, and the east–west span being 20°. The total area of suitable habitat for *H. cunea* was 118 × 10^4^ km^2^, accounting for 12% of the total land area of China. Thus, *H. cunea* is an invasive insect group with an extremely wide distribution and poses a high risk.

### 3.4 Future changes in suitable habitat area

**FIGURE 5:**
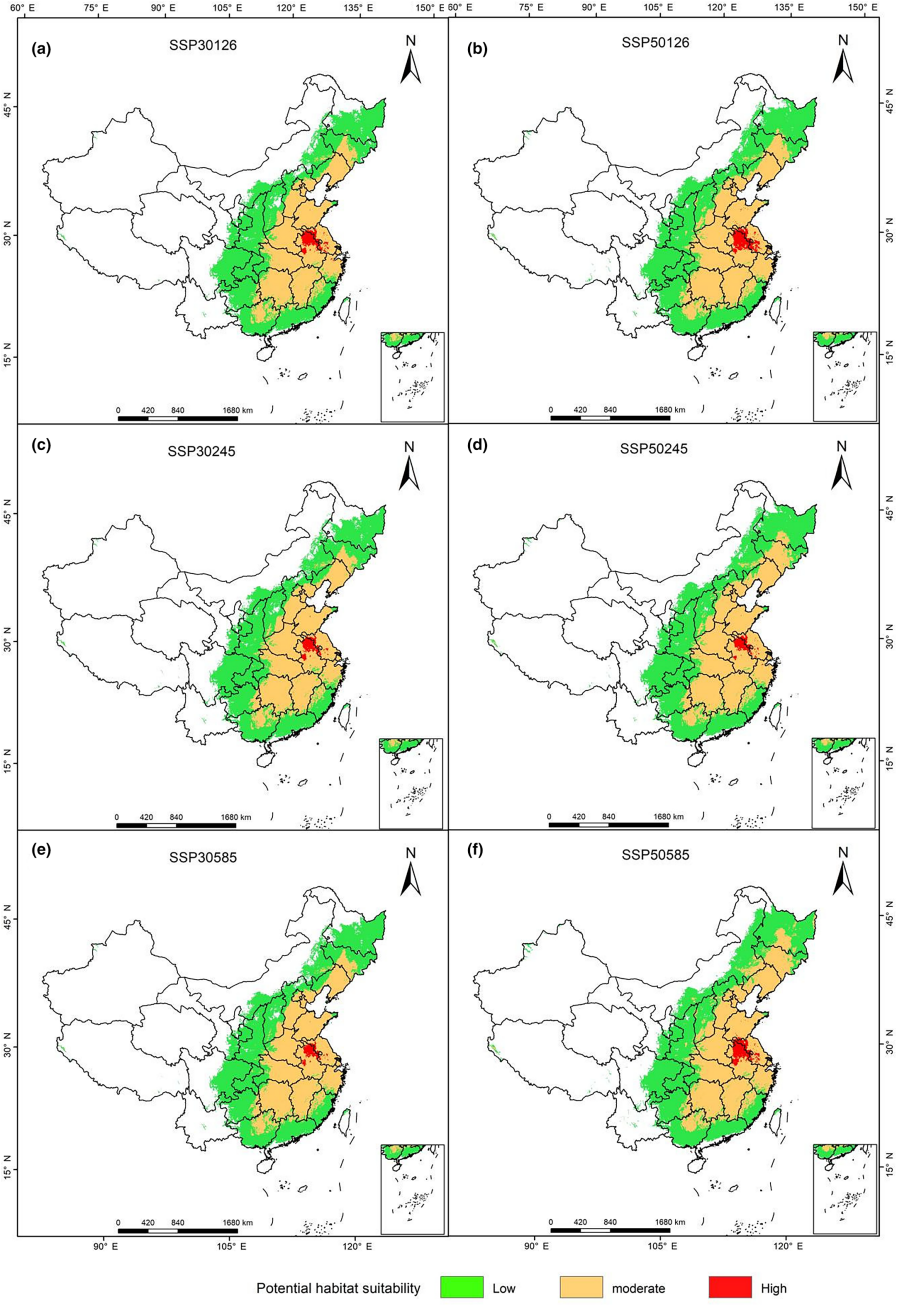
The spatial pattern changes of *Hyphantria cunea* in different periods. In the figure, 30 and 50 represent the average values for 2021 to 2040 and 2041 to 2060, respectively, and SSP126, 245, and 585 represent the three scenarios of low, medium, and high climate change intensity, respectively.

In all future climatic scenarios, the suitable habitats of *H. cunea* in China were predicted to increase (Table 2); the increased area generally exceeded 230 × 10^4^ km^2^, the rate of increase exceeded 200%, and the increased area was more than 1/3 of the entire land area of China. There was a clear trend indicating that the species would spread from existing occurrence areas to the surrounding areas. The newly added suitable habitats were located mainly in Jilin, Heilongjiang, and southern Inner Mongolia in northeastern China; Fujian, Guangdong, and Guangxi in the coastal areas; and Hunan and Jiangxi Provinces along the Yangtze River (Figure 5).

**TABLE 2:** Changes in the distribution area of *Hyphantria cunea* in different periods and different scenarios.

Period	Climate scenario	Habitat area (×10^4^ km^2^)	Gain (×10^4^ km^2^)	Percentage gain (%)	Low habitat suitability (×10^4^ km^2^)	Moderate habitat suitability (×10^4^ km^2^)	High habitat suitability (×10^4^ km^2^)
Current		118			48	33	37
2021–2040	SSP126	355	237	200.85	202	147	6
	SSP245	357	239	202.54	202	150	5
	SSP585	357	239	202.54	202	151	4
2041–2060	SSP126	377	259	219.49	207	162	8
	SSP245	382	264	223.73	211	167	4
	SSP585	394	276	233.90	208	179	7

The effect of different climate scenarios on the increased area of suitable habitat for *H. cunea* was very small. Generally, with increasing carbon emissions and time, the suitable area for *H. cunea* will increase further, but the area of its highly suitable habitat will decrease. In the middle of this century, SSP585 was the most favorable scenario for promoting the spread of *H. cunea*, with the area of moderately and highly suitable habitat increasing by 116 × 10^4^ km^2^, which is indicated by the changes in the red and yellow areas in the figure.

## 4 DISCUSSION

### 4.1 The effect of species distribution points on the model

Species distribution points reflect the species' niche requirements, which are at the core of the SDM equilibrium hypothesis (Araújo & Peterson, 2012). Several previous studies showed that using a relatively large number of species distributions for modeling can effectively reduce the overfitting produced by ENMs (Petitpierre et al., 2017; Roberts et al., 2017). In this study, the evaluation showed that the completeness of species distribution points was crucial to the accuracy of the results, and an SDM prediction using only *H. cunea* distribution data from invaded areas had a maximum accuracy of only 53.04%. On the one hand, the results suggested that ecological niche conservatism is sometimes not applicable to invasive insects (Atwater & Barney, 2021; Gallien et al., 2012); on the other hand, the results also showed that after a long period of invasion (e.g., 27 years of *H. cunea* invasion in China (1979–2006)), invasive insects still have high potential to expand into new areas (Liu et al., 2022; Václavík & Meentemeyer, 2012). Therefore, for future research on suitable habitats for invasive insects, we suggest using the SDM for prediction after obtaining information on the sites of origin and invasion, which will greatly improve the accuracy of prediction.

### 4.2 The effect of model selection on the prediction results

There are differences in the prediction results of different SDMs for different species (Srivastava et al., 2021). A more obvious problem is that for different species, different methods are required to obtain the optimal result instead of repeating the study with the same method, which is particularly relevant to the use of an SDM (Qiao et al., 2015; Segurado & Araújo, 2004). Our results show that the EM predicts *H. cunea* better than MaxEnt when using the same data, which is consistent with results obtained by Marmion et al. (2009) in Finland and by Breiner et al. (2015) on plants. A significant advantage of an EM is that the EM can effectively combine multiple models to capture the true “signal” of species niches and reduce some “noise” caused by errors and uncertainties in data and model structure (Dormann et al., 2018; Hao et al., 2019), and this advantage is particularly applicable to newly invasive organisms, especially those whose ecological relationships are not yet clear.

### 4.3 Limitations of ENMs on invasive insects

Using global datasets and an EM, the niche approach achieved good results for both long‐term (12‐year) and short‐term (3‐year) predictions of *H. cunea*, but there are still many limitations affecting the application of this method to invasive insects. One consideration is that other comprehensive global datasets, such as that of *H. cunea*, may not be available for other invasive insects. Currently, more than 7700 species of invasive insects have been recorded worldwide (Zhao et al., 2022). Among them, fewer than a few hundred species have been studied extensively (Kenis et al., 2009). As mentioned earlier, species distribution points are critical to prediction accuracy, and the inability to obtain comprehensive species distribution points could directly constrain the application of ENMs to study these species. Another main factor is the prediction accuracy. Since the destructive power of invasive insects is incredibly high, from the perspective of management, it is often necessary to have a 100% understanding of the possible occurrence areas. However, when insects enter a new environment, they undergo a complex series of adaptations to the environment (Catford et al., 2022; Melbourne & Hastings, 2009), which makes it difficult for the ENM to predict the distribution in the new environment based on the original mechanisms, resulting in certain errors in the results.

### 4.4 Recommendations for *Hyphantria cunea* management

There is no doubt that the increase in the area of suitable habitat poses great challenges to the management of *H. cunea*, although this does not mean that *H. cunea* will certainly invade these areas. This study showed that the areas of invasion are often overestimated. However, a large increase in area could lead to many uncontrollable risks; for example, areas in which *H. cunea* cannot survive the winter could become viable, quarantine measures may need to be expanded, and species could change regularly (Pyšek et al., 2020; Roques et al., 2016). Therefore, it is necessary to strengthen the management of *H. cunea* to prevent its invasion of areas that are not currently invaded but could be suitable in the future to reduce losses (Bradshaw et al., 2016). Shanxi Province in China may provide a good example. In many predictions, Shanxi Province is a highly suitable habitat for *H. cunea*, but since 1979, there has been no occurrence of *H. cunea*. Through communication with the local management departments, two management differences were found, which may be the key to avoiding *H. cunea* invasion:

1. High levels of indigenous protectionism. In the urban greening of Shanxi Province in recent decades, out of concern for the protection of local seedling production, only seedlings grown in Shanxi Province have been used. This measure eliminates the introduction of *H. cunea* pupae by seedlings with soil, which is the main way *H. cunea* spreads over long distances.
2. Extremely harsh quarantine measures. The eastern and southern parts of Shanxi Province are areas with large occurrences of *H. cunea*. Local governments have adopted extremely harsh management measures for the surrounding areas. Once the insects are found, they are immediately sent for testing, and extremely high penalties are imposed on departments that fail to detect and dispose of them in a timely manner.

The above experience suggests that avoiding invasion from the source may be the best strategy for blocking the spread of invasive organisms. For the future management of *H. cunea*, we recommend the following: (i) strengthening source control and adopting origin quarantine for seedling transport to prevent the spread of *H. cunea*; (ii) enhancing monitoring so that *H. cunea* can be detected in the early stage of invasion; and (iii) for key ecological locations and parks, adopting an island strategy to prohibit the entry of foreign transmission vectors and cut off the transmission channels of *H. cunea* at the source.

## AUTHOR CONTRIBUTIONS

**Xuanye Wen:** Conceptualization (equal); data curation (equal); formal analysis (equal); methodology (equal); software (equal); validation (equal); visualization (equal); writing – original draft (equal). **Guofei Fang:** Conceptualization (equal); formal analysis (equal); writing – original draft (equal). **Shouquan Chai:** Methodology (equal); validation (equal). **Chuanjie He:** Formal analysis (equal); writing – original draft (equal). **Shouhui Sun:** Conceptualization (equal); supervision (equal); writing – original draft (equal). **Guanghua Zhao:** Formal analysis (equal); software (equal); writing – original draft (equal). **Xiao Lin:** Conceptualization (equal); funding acquisition (equal); supervision (equal); writing – review and editing (equal).

## CONFLICT OF INTEREST STATEMENT

None declared.

## Supporting information


Data S1.

## Data Availability

The data that support the findings of this study are openly available in Dryad. DOI: 10.5061/dryad.jq2bvq8f5.
