# Supplementary material for: Can ecological niche models be used to accurately predict the distribution of invasive insects? A case study of Hyphantria cunea in China
Source: Ecol Evol. 2024 Mar 14;14(3):e11159. doi: 10.1002/ece3.11159 (PMC10940052; doi:10.1002/ece3.11159)
Supplement: Supplementary file 1 — Data S1. [file ECE3-14-e11159-s001.docx]

Supporting information

**Can ecological niche models be used to accurately predict the distribution of invasive insects? A case study of *Hyphantria cunea* in China**

Xuanye Wen^a^, Guofei Fang^a^, Shouquan Chai^a^, Chuanjie He^a^, Shouhui Sun^b*^, Guanghua Zhao^c*^, Xiao Lin^a*^

^a^Center for Biological Disaster Prevention and Control, National Forestry and Grassland Administration, Shenyang 110031, China.

^b^College of Forestry, Shenyang Agricultural University, Shenyang 110866, China.

^c^College of Life Sciences, Shanxi Normal University, Taiyuan 030000, China.

*Corresponding author

ssh1@syau.edu.cn (Shouhui Sun); [zgh3051@1](mailto:fmyan@henau.edu.cn)63.com (Guanghua Zhao)[;](mailto:sfzzlfc@163.com;) sfzzlfc@163.com (Xiao Lin)

Table S1. Environmental variables for modeling.

| Type | Variables | Description | UNITS |
| --- | --- | --- | --- |
| Bioclimatic Variables | Bio1 | Annual Mean Temperature | ℃ |
|  | Bio4 | Temperature Seasonality | 1 |
|  | Bio5 | Max Temperatur | ℃ |
|  | Bio6 | Min Temperature of Coldest Month | ℃ |
|  | Bio11 | Mean Temperature of Coldest Quarter | ℃ |
|  | Bio12 | Annual Precipitation | mm |
|  | Bio14 | Precipitation of Driest Month | mm |
|  | Bio16 | Precipitation of Wettest Quarter | mm |
|  | Bio17 | Precipitation of Driest Quarter | mm |
| Terrain | ELEV | Elevation | m |

**
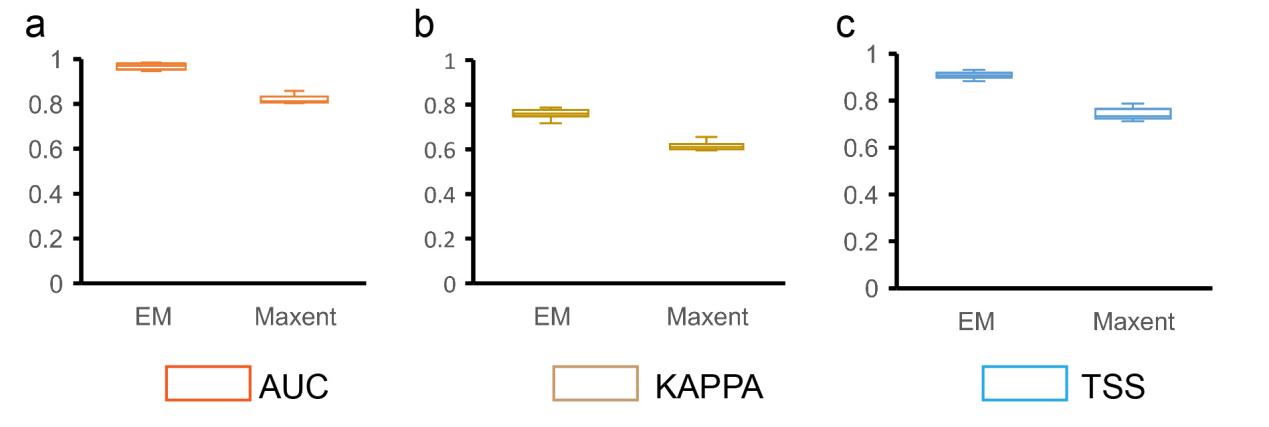
Fig. S1.** Evaluation scores of ensemble model and Maxent.
